# Laparoscopic and Robotic Total Mesorectal Excision in the Treatment of Rectal Cancer. Brief Review and Personal Remarks

**DOI:** 10.3389/fonc.2014.00098

**Published:** 2014-05-06

**Authors:** Paolo Pietro Bianchi, Wanda Petz, Fabrizio Luca, Roberto Biffi, Giuseppe Spinoglio, Marco Montorsi

**Affiliations:** ^1^Unit of Minimally Invasive Surgery, European Institute of Oncology, Milan, Italy; ^2^Unit of Abdominal Integrated Surgery, European Institute of Oncology, Milan, Italy; ^3^Division of Abdomino-Pelvic Surgery, European Institute of Oncology, Milan, Italy; ^4^Department of General and Oncologic Surgery, Azienda Ospedaliera SS Antonio e Biagio, Alessandria, Italy; ^5^Division of General Surgery, Istituto Clinico Humanitas, School of Medicine, University of Milan, Rozzano, Italy

**Keywords:** rectal cancer, total mesorectal excision, robotic surgery, neoadjuvant therapy, laparoscopic surgery

## Abstract

The current standard treatment for rectal cancer is based on a multimodality approach with preoperative radiochemotherapy in advanced cases and complete surgical removal through total mesorectal excision (TME). The most frequent surgical approach is traditional open surgery, as laparoscopic TME requires high technical skill, a long learning curve, and is not widespread, still being confined to centers with great experience in minimally invasive techniques. Nevertheless, in several studies, the laparoscopic approach, when compared to open surgery, has shown some better short-term clinical outcomes and at least comparable oncologic results. Robotic surgery for the treatment of rectal cancer is an emerging technique, which could overcome some of the technical difficulties posed by standard laparoscopy, but evidence from the literature regarding its oncologic safety and clinical outcomes is still lacking. This brief review analyses the current status of minimally invasive surgery for rectal cancer therapy, focusing on oncologic safety and the new robotic approach.

## Introduction

The main innovations over the last decades for the treatment of locally advanced rectal cancer have been multimodal preoperative treatments with preoperative radiochemotherapy and the optimization of surgical technique with the introduction of TME (1, 2).

Total mesorectal excision changed the technique of rectal resection and confirmed the importance of maintaining the integrity of the mesorectal fascia to improve oncologic outcomes. As a result, in some series, TME surgery has been demonstrated to decrease the rate of local recurrence (LR) to <10%, when compared to conventional dissection (3).

Nowadays, TME surgery preceded by neoadjuvant chemoradiation is considered as the standard of care for locally advanced rectal cancer (4). The opportunity to perform a minimally invasive TME is correlated to the clinical benefits demonstrated by minimally invasive surgery (MIS) (5). Laparoscopic colectomy is a feasible and oncologically safe procedure with an increasing diffusion, but minimally invasive TME is less adopted because of its technical difficulties (6).

Robotic surgery is a new emerging technique that seems to overcome some difficulties of standard laparoscopic approach in the pelvis, and its use in rectal cancer is under evaluation (7, 8).

This mini review summarizes the current role of MIS in rectal resections for cancer also evaluating the recent developments of robotic technique.

## Laparoscopic Surgery in Rectal Cancer

The widespread adoption of MIS in several surgical procedures is correlated with some short-term better clinical outcomes achievable by this technique when compared to traditional open surgery.

In the surgical treatment of cancer, the diffusion of MIS has been slower than in other benign diseases, due to the necessity to confirm the oncologic safety of the technique (9).

Nowadays, long-term oncologic results of laparoscopic vs. open surgery for colon cancer have been established by several randomized clinical trials (10–12).

These results have overcome the initial concern about oncologic safety and port-site recurrence of MIS for cancer (13, 14), but technical difficulties of laparoscopic resection and the consequent long learning curve have limited its dissemination outside specialized centers (15–17).

Technical difficulties are increased in laparoscopic rectal resection because of the anatomy of the pelvis, where it is difficult to manipulate the straight laparoscopic instruments. Therefore, rectal MIS is even less widespread than laparoscopic colectomy (18).

The diffusion of laparoscopic colonic surgery has increased in recent years by specific educational and training programs, but mainly in academic and high-volume hospitals (18, 19). The most frequently performed procedures are the less difficult, such as sigmoidectomy for benign diseases and right colectomies with extracorporeal anastomosis (6). In the United States, laparoscopic rectal resection did not make up 20% of the overall rectal resections and its conversion rate to open surgery is still high (46.2%), without any significant improvement in recent years (18). For this reason, still in 2012, the National Comprehensive Cancer Network guidelines for treatment in rectal cancer recommended the use of laparoscopy for rectal cancer treatment only within a study protocol and in highly specialized centers (4).

Three randomized controlled trials evaluated feasibility and oncologic safety of laparoscopic TME (20–22). The first report of the CLASICC trial comparing laparoscopic to open surgery and including rectal resections (20), raised a concern about an increased positivity of the circumferential resection margin (CRM) among patients in the laparoscopic group, but this result was not translated into any significant difference compared to open surgery in terms of survival rates after 6 years of follow-up. (23). The COREAN trial (21) compared laparoscopy to open surgery in rectal cancer after neoadjuvant chemoradiotherapy, and although participating surgeons had greater experience in laparoscopic surgery than did surgeons participating in the CLASICC trial, the differences in percentage of CRM positivity were similar in the two groups: 4.1% in the open group and 2.9% in the laparoscopic group. The recently published COLOR II study (22), also comparing laparoscopic to open surgery in rectal resections, did not reveal any significant difference between the two procedures in terms of morbidity, mortality, and complication rates, and confirmed the benefits of the minimally invasive approach as less blood loss, more rapid recovery of bowel function, and shorter hospital stay.

Although the participating surgeons were all experts in laparoscopic surgery, the conversion rate to open surgery was still high (16%), confirming the technical challenges of laparoscopic rectal surgery. Interestingly, in contrast to the CLASICC trial, in the COLOR II study, a proportion of higher CRM positivity was found in the open rather than in the laparoscopic arm, in the distal rectal cancer subgroup (22 vs. 9%, *p* = 0.014) (Table 1). However, the number of patients is too low to consider these data conclusive and the trial has a possible bias of selection, because patients with a T4 or a T3 tumor within 2 mm of endopelvic fascia were excluded (22).

**Table 1:** **Comparison of circumferential resection margin positivity in open and laparoscopic total mesorectal excision (TME)**.

	Overall (%)			AR (%)			APR (%)		
	Open	LAP	*p*	Open	LAP	*p*	Open	LAP	*p*
CLASICC (20)	14	16	0.8	6	12	0.19	26	20	1.0
COREAN (21)	4.1	2.9	0.7	3.4	2.7	1.0	8.3	5.3	1.0
COLOR II (22) distal rectal cancer	10	10	0.8	22	9	0.01	25	8	0.003

*AR, anterior resection; APR, abdominoperineal resection; LAP, laparoscopic resection; *p, p* value*.

The conversion of a laparoscopic to an open procedure is a critical issue, since it has been demonstrated to lead to an increase in complication rate, length of hospital stay, overall costs, and in some studies also to have a negative impact on LR and overall survival rates (12, 24).

Our experience in laparoscopic rectal resection as a high-volume surgical center is comparable to that reported in the literature. In a prospective analysis of more than 100 laparoscopic rectal resections with a mean follow-up of 35.8 months, oncologic results were not inferior to those of standard open surgery, but the technique was considered demanding, because of the high percentage of conversion rate to open (18.7%) and the prolonged operating time (mean operating time, 278 min). The conclusion of that study was that laparoscopic rectal resection should be performed in specialized centers by teams experienced in laparoscopic surgery (17).

## The Emerging Role of Robotic-Assisted Surgery

Robotic surgery is an emerging minimally invasive technique. The robotic system is composed of three integrated elements: a surgeon console, a patient-side cart with interactive robotic arms connected to the surgical instruments and a video tower with the system processors, and a high-definition three-dimensional vision system. The optical system provides a high definition, three-dimensional vision, and surgical instruments are provided for seven degrees of freedom and for a range of motion greater than the human wrist; this enables extremely fine and precise manual dexterity. Therefore, robotics seems to have the potential to overcome some of the technical difficulties of traditional laparoscopic surgery, allowing high-quality maneuvers to be performed in narrow spaces such as the pelvic cavity, and using a third arm instrument as a fixed retractor, improving vision and stability in restricted spaces. The results available on robotic rectal resections are limited, originating mainly from single-center experiences, but the interest of the scientific community is high as demonstrated by the several meta-analyses already published despite the lack of evidence (25–29). In all of these studies, the only significant data was that robotic surgery resulted in a lower percentage of conversion to open surgery, compared to the laparoscopic groups. Regarding short-term clinical and oncologic outcomes, no significant differences were found between laparoscopy and robotic surgery, as reported in Table 2 and Table 3. Few studies have compared robotic surgery to standard treatment of open resection, and in these studies robotic surgery resulted oncologically safe in terms of length of specimen, resection margins, and number of lymph nodes harvested (37, 38).

**Table 2:** **Oncologic results of laparoscopic and robotic surgery for rectal cancer**.

	Harvested lymph nodes (*n*)		*p*	Distal resection margin (cm)		*p*	Positive CRM (%)		*p*
	ROB	LAP		ROB	LAP		ROB	LAP	
Park et al. (30)	17.3	14.2	0.06	2.1	2.3	ns	4.9	3.7	0.5
Kim and Kang (31)	14.7	16.6	ns	2.7	2.6	0.09	3	2	ns
Kwak et al. (32)	20	21	0.7	2.2	2.8	0.8	1.7	0	>0.9
Baek et al. (33)	13	16	0.07	3.6	3.8	0.6	2.4	4.9	1
Bianchi et al. (34)	18	17	0.7	2	2	1.0	0	4	0.9
Baik et al. (35)	18.4	18.7	0.8	4	3.6	0.4	7	8	0.7
Patriti et al. (36)	10.3	11.2	>0.05	2.1	4.5	>0.05	0	0	ns

*Meta analysis [Memon et al. (25)]*.

*CRM, circumferential resection margin; ROB, robotic resection; LAP, laparoscopic resection; *p, p* value*.

**Table 3:** **Clinical results of laparoscopic and robotic surgery for rectal cancer**.

	Conversions (%)		*p*	Hospital stay (days)		*p*	Complications (%)		*p*
	ROB	LAP		ROB	LAP		ROB	LAP	
Park et al. (30)	0	0	1	9.9	9.4	0.5	29.3	23.2	0.4
Kim and Kang (31)	2	3	1	11.7	14.4	0.006	20	27	0.4
Kwak et al. (32)	0	3.4	0.4	NA	NA		32	27	ns
Baek et al. (33)	7.3	22	0.116	6.5	6.6	0.8	22	27	1
Bianchi et al. (34)	0	4	NA	6.5	6	0.4	16	24	0.5
Baik et al. (35)	0	10.5	0.013	5.7	7.6	0.001	10.7	19.3	0.025
Patriti et al. (36)	0	19	<0.05	11.9	9.6	>0.05	30.6	18.9	>0.05

*Meta analysis [Memon et al. (25)]*.

*ROB, robotic resection; LAP, laparoscopic resection; *p, p* value; NA, not available; ns: not significant*.

One of the main concerns about robotic technology is the high costs of the purchase and maintenance of the equipment. Baek et al. (39) showed increased costs in robotic rectal resection compared to those in the standard laparoscopic procedure, with a significantly lower hospital profit in the robotic group.

Another emerging problem is the appropriate use of the technology by low volume centers/surgeons, in fact a higher number of complications are reported by Keller et al. (40) in the low volume users when compared to middle- and high-volume centers and surgeons.

Recent studies have demonstrated a superiority of robotic rectal resection in recovery of urinary voiding and sexual function (41, 42), but these results need to be confirmed by larger randomized studies.

At present, two multicentre studies comparing robotic to laparoscopic TME (ROLARR and COLRAR) are ongoing and results are awaited to better define the role of robotic rectal surgery (43, 44).

In 2008, we launched a robotic program in rectal cancer, extended to anesthesiologists and operating room nurses. The only criterion of choice between laparoscopic and robotic-assisted resection was the availability of the robotic system. The first 25 robotic-TME procedures were compared with 25 well-matched laparoscopic procedures, and the results were similar in terms of clinical and oncologic outcomes (34). In the robotic group, no patients were converted to open, while in the laparoscopic group, the conversion rate was 4% (1/25). Operating time was similar in the two groups (240 min laparoscopic TME, 237 min robotic-TME) and was not modified in the robotic-TME group during time (Table 4).

**Table 4:** **Clinical and oncologic outcomes of 50 minimally invasive total mesorectal excision (TME)**.

	ROB	LAP	*p*
Complications (%)	16	24	0.5
Median (range) operating time (min)	240 (170–420)	237 (170–545)	0.2
Conversions *n* (%)	0 (0)	1 (4)	
Median (range) first bowel movements (days)	2 (1–7)	3 (1–4)	0.5
Median (range) hospital stay (days)	6.5	6	0.4
Median (range) lymph nodes/patient (*n*)	18	17	0.7
Median (range) distal resection margin (cm)	2	2	1.0
CRM positivity *n* (%)	0/25 (0)	1/25 (4)	0.9

*Bianchi et al. (34)*.

*ROB, robotic resection; LAP, laparoscopic resection; *p, p* value; CRM, circumferential resection margin*.

## Discussion and Personal Remarks

Minimally invasive surgery is considered one of the most important innovations in surgical technique for the last 25 years. One of the goals of surgical oncology is the possibility to reduce the invasiveness of surgery maintaining or further improving the results of traditional open surgery. Therefore, minimally invasive techniques have been applied to oncologic surgery since the end of 90s, on the basis of the good clinical results obtained by laparoscopic surgery in some benign diseases (45, 46).

The first colonic resection was described in 1991 (47) and a lot of concern raised on the oncologic safety of the procedure, in terms of length of the specimen removed and number of lymphnodes harvested (48). Nevertheless, the way of minimally invasiveness seemed to the great majority of surgeons to be the right way to follow and since then several RCTs have been published comparing laparoscopic to open colectomy (20, 49–52).

Since the first study of Lacy et al. published in 2002 (53), all the following trials comparing laparoscopic to open colectomy demonstrated the oncologic safety of laparoscopy, then confirmed also as long-term survival rates (10–12).

Laparoscopy was further associated to some short-term advantages as: less pain, less intraoperative blood loss, shorter duration of postoperative ileus, shorter hospital stay, improved pulmonary function, decrease of total and local morbidity, quicker recovery, and less inflammatory factors activation (5, 54).

On the basis of the results of these RCTs, the conclusion of a Cochrane review in 2005 was that “laparoscopic approach should be preferred in patients suitable for this approach to colectomy” (5). Nevertheless, the diffusion of laparoscopic colectomy has been slower and more difficult than supposed, probably due to the long learning curve and to the absence of well-planned educational programs.

Only in the last few years, the trend was slightly inverted with an increase of laparoscopic surgery in colon resection, even if mainly in selected cases and in specialized centers (19).

The role of a planned educational program is crucial to improve the safety of the procedure and to increase its spread, as reported by the results of the Dutch Surgical Colorectal Audit (55), although in this report the percentage of conversions to open surgery is still 15%.

Regarding minimally invasive rectal cancer surgery, we are a step back to laparoscopic colectomy, either in terms of diffusion of the procedure either in terms of level of evidence (56).

The available RCTs demonstrate the feasibility, safety, and oncologic adequacy of minimally invasive TME, but long-term high evidence data come essentially from the CLASICC study, where the majority of participating surgeons were low experienced (12).

The learning curve of minimally invasive TME is longer and steeper than for laparoscopic colectomy and the percentage of conversions to open surgery is still high, confirming the technical difficulties of the procedure and still the necessity of an accurate selection of patients (57). For example, one of the most difficult steps of the procedure is distal resection of the rectum, and robotic assistance could be helpful in the exposure of the distal pelvis, facilitating the stapler positioning for transection.

For these reasons, the diffusion of laparoscopic TME in the last years has been low and without a trend to increase. Nevertheless, the post operative short-term advantages of the minimally invasive approach are confirmed, as for laparoscopic colectomies, therefore, an increasing diffusion of minimally invasive TME should be desirable.

Robotics is a technology, which helps minimally invasive pelvic surgery and probably also rectal resections. It is our opinion that robotic assistance with virtual simulators or dual console proctoring systems could be important devices to facilitate the adoption of minimally invasive rectal surgery.

Although the results available on robotic surgery are still few, robotic assistance seems to reduce the percentage of conversions to open surgery among expert surgeons and is promising as a method to attenuate the learning curve of a well-conducted TME (58). At the moment, the robotic system has higher costs than laparoscopy and its use should be planned within the remit of clearly defined educational program, preferably in a hospital conducting middle/high volumes of MIS and colorectal procedures, in order to avoid an increase in complication rates.

## Conflict of Interest Statement

The authors declare that the research was conducted in the absence of any commercial or financial relationships that could be construed as a potential conflict of interest.
